# Evaluation of farmers friendly IPM modules for the management of fall armyworm, *Spodoptera frugiperda* (JE Smith) in maize in the hot semiarid region of India

**DOI:** 10.1038/s41598-024-57860-y

**Published:** 2024-03-26

**Authors:** Mandla Rajashekhar, Banda Rajashekar, Thalla Prabhakar Reddy, Keerthi Manikyanahalli Chandrashekara, Kalisetti Vanisree, Kommagoni Ramakrishna, Vanam Sunitha, Ongolu Shaila, Eetela Sathyanarayana, Shahanaz, Somireddy Srinivasa Reddy, Adhi Shankar, Afifa Jahan, Padala Vinod Kumar, Maligi Jagan Mohan Reddy

**Affiliations:** 1 Entomology Department, Institute of Biotechnology, Professor Jayashankar Telangana State Agricultural University (PJTSAU), Hyderabad, Telangana India; 2 Krishi Vigyan Kendra, Palem, Professor Jayashankar Telangana State Agricultural University (PJTSAU), Hyderabad, Telangana India; 3 Division of Crop Protection, ICAR- Indian Institute of Horticulture Research, Hesaraghatta, Bengaluru, India; 4 Maize Research Centre, Professor Jayashankar Telangana State Agricultural University (PJTSAU), Hyderabad, Telangana India; 5 All India Network Project On Vertebrate Pest Management, Professor Jayashankar Telangana State Agricultural University (PJTSAU), Hyderabad, Telangana India; 6 Department of Soil Science and Agricultural Chemistry, Agricultural College, Palem, Professor Jayashankar Telangana State Agricultural University (PJTSAU), Hyderabad, Telangana India; 7 College of Horticulture, Mojerla, Sri Konda Laxman Telangana State Horticultural University, Rajendranagar, Hyderabad, India; 8 Seed Research Technology Centre, Professor Jayashankar Telangana State Agricultural University (PJTSAU), Hyderabad, Telangana India; 9 ICAR- RCER, Research Centre for Makhana, Darbhanga, Bihar 846005 India; 10 Extension Education Institute (Southern Region), Professor Jayashankar Telangana State Agricultural University (PJTSAU), Hyderabad, Telangana India

**Keywords:** Fall armyworm, Invasive pests, IPM, *Metarhizium anisopliae*, Pheromone traps, *Spodoptera frugiperda*, Technology gap, Ecology, Plant sciences, Zoology, Environmental sciences

## Abstract

Invasive alien species (IAS) pose a severe threat to global agriculture, with their impact projected to escalate due to climate change and expanding international trade. The fall armyworm (FAW), *Spodoptera frugiperda* (J. E. Smith), a native of the Americas, has rapidly spread across various continents, causing significant damage to several food crops, especially maize. Integrated pest management (IPM) programs are vital for sustainable FAW control, combining multiple strategies for sustainable results. Over three consecutive years, 2019–20, 2020–21 and 2021–22, the field demonstrations were conducted in semiarid regions of India, testing a four-component IPM approach viz., pheromone traps, microbial, botanicals and ETL based applications of insecticides against farmers' practices (sole insecticide application). IPM implementation led to substantial reductions in FAW infestation. Furthermore, egg mass and larvae infestations were significantly lower in IPM-adopted villages compared to conventional practices. Pheromone-based monitoring demonstrated a consistent reduction in adult moth populations. The lowest technology gap (10.42), extension gap (8.33) and technology index (12.25) was recorded during 2020–21. The adoption of IPM led to increased maize yields (17.49, 12.62 and 24.87% over control), higher net returns (919, 906.20 and 992.93 USD), and favourable benefit–cost ratios (2.74, 2.39 and 2.33) compared to conventional practices respectively during 2019–20, 2020–21 and 2021–22. The economic viability of IPM strategies was evident across three consecutive years, confirming their potential for sustainable FAW management in the semiarid region of India. These strategies hold promise for adoption in other parts of the world sharing similar climatic conditions.

## Introduction

Invasive alien species (IAS) constitute a serious threat to agricultural products, and this threat is expected to grow in response to climate change and growing global trade^1^. The IAS also supplant native species, harm biodiversity, and alter ecosystems, resulting in significant economic losses^1–4^. The fall armyworm (FAW), *Spodoptera frugiperda* (J. E. Smith) (Lepidoptera: Noctuidae), is a native species to the tropical and subtropical areas of the Americas^5^. After being first identified in West Africa^6^ it quickly spread to sub-Saharan Africa^7^ the Asian subcontinent^8–10^. The FAW has a wide host range of over 353 plants from 76 families, primarily from the Poaceae (106), Asteraceae (31), and Fabaceae (31)^11,12^. The infestations of FAW have been shown to reduce maize yields by 15–73%^9^. Due to the high consumption of these cereal crops, mainly maize, in smallholder diets, FAW could substantially impact global food security. Further the damage on maize, may aggravates the dependent industries like bio-ethanol, poultry, and animal husbandry^9^.

The management of FAW is challenging mainly because of higher reproduction potential, polyphagous feeding habitat, strong flight capability, absence of diapause, potential displacement of rival species and evolved resistance to an array of pesticide classes. In addition, mature larvae's cryptic eating habits frequently render chemical insecticides inefficient. The farmers have attempted to control this damaging pest using several physical, chemical, and biological methods, but most of these have been ineffective when used individually^13^. So, for long-term FAW control, Integrated Pest Management (IPM) programmes comprising a variety of supplementary approaches are required. IPM is an ecosystem-based strategy that focuses on long-term prevention of pests or their damage through a combination of techniques such as biological control, habitat manipulation, modification of cultural practices, and use of resistant varieties. For early identification of pest, and to make timely and effective management decisions, pheromone-based monitoring, surveillance, and scouting using flying moths are crucial and essential tools^7,14^.

The effectiveness of pest control techniques in mitigating crop losses is contingent on farmers' comprehension, attitudes, and practices related to pest management. Therefore, it is hypothesized that evaluating farmers' knowledge concerning insect pests, yield damage, and efficient management strategies through surveys is imperative. Additionally, current agricultural pest management practices require a robust scientific foundation, and the rationale behind farmers' adoption of these practices may be uncertain. Farmers have the potential to employ crop rotation, intercropping, and resistant crop varieties to minimize infestations^13^. Biological control, involving the use of natural enemies like parasitoids, predators, and entomopathogens, can significantly reduce pest populations when integrated with compatible pest management techniques^8^. Moreover, the controlled and prudent use of insecticides, coupled with appropriate safety measures to prevent adverse impacts on human health and the environment, may not be environmentally harmful. These methods aim to enhance the resilience of the agro ecosystem and reduce the pest's reproductive potential and dispersal. Early inspection and monitoring of crops with pheromone traps, have proven effective in managing the fall armyworm in the northeastern states. It is imperative to provide periodic awareness training for maize growers and enhance the capacity of extension officers and input dealers in early scouting, surveillance, and monitoring of FAW incidence to achieve similar success in other regions. The hypothesis regarding Integrated Pest Management (IPM)-based strategies for FAW suggests that such approaches can efficiently control the pest and enhance crop productivity compared to conventional pesticide applications. IPM strategies encompass various techniques including cultural, biological, mechanical, and behavioral methods such as crop rotation, resistant varieties, natural enemies, pheromone traps, and bio-pesticides. In India, researchers have explored different modules, including bio-intensive, IPM, and synthetic insecticide-based practices for managing *S. frugiperda*^15^. While initially, the exclusive use of insecticides proved effective, *S. frugiperda* has since developed resistance to commonly used insecticides like cypermethrin^16^, lambda cyhalothrin^17^ and profenophos^18,19^ rendering them ineffective at the recommended field doses. Similarly, IPM results have been satisfactory, it's noted that these approaches may not be universally applicable across all agro-ecological regions of India due to variations in inputs and challenges in availability. Successful IPM implementation requires a participatory approach involving farmers, extension agents, researchers, and policymakers to ensure adoption and sustainability. Given the location-specific nature of IPM strategies influenced by prevailing weather conditions, it's crucial to develop and validate location-specific models for effective FAW management in maize crops. In order to validate the hypothesis, interviews were carried out with farmers to discern their preferred FAW control methods, based on which compatible components of IPM were selected and evaluated under farmers' field conditions.

## Results

**Figure 1:**
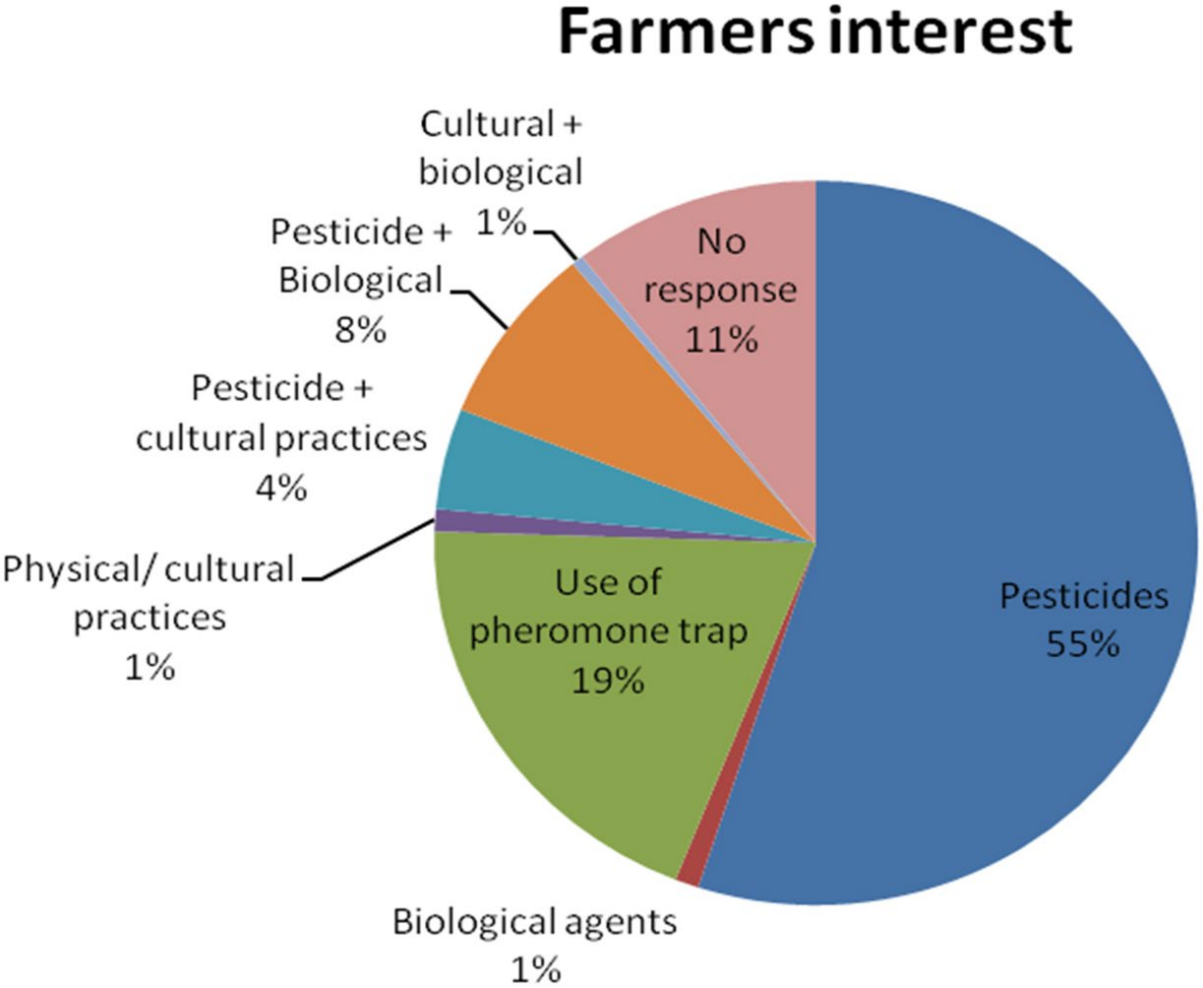
Preference of farmers towards different management practices for FAW management.

A total of 200 farmers were interviewed for their preference towards the choice of control methods for FAW management. Among the options listed, pesticides were the most preferred choice, with 55% of the farmers expressing interest in using them for FAW management. Biological pest control, which utilizes natural enemies of *S. frugiperda*, garnered minimal interest, with only 1% of farmers showing a preference for this approach (Fig. 1). Interestingly, use of pheromone traps captured the attention of 19.5% of the farmers. Some farmers (1%) expressed interest in physical or cultural practices. Combining pesticides with cultural practices or biological agents gained interest from 4.5 and 8% of the farmers, respectively. Surprisingly, only 0.5% of the farmers showed interest in combining cultural practices with biological agents. Additionally, 10.5% of the farmers did not respond, suggesting a lack of awareness or uncertainty regarding the various FAW control methods.

**Figure 2:**
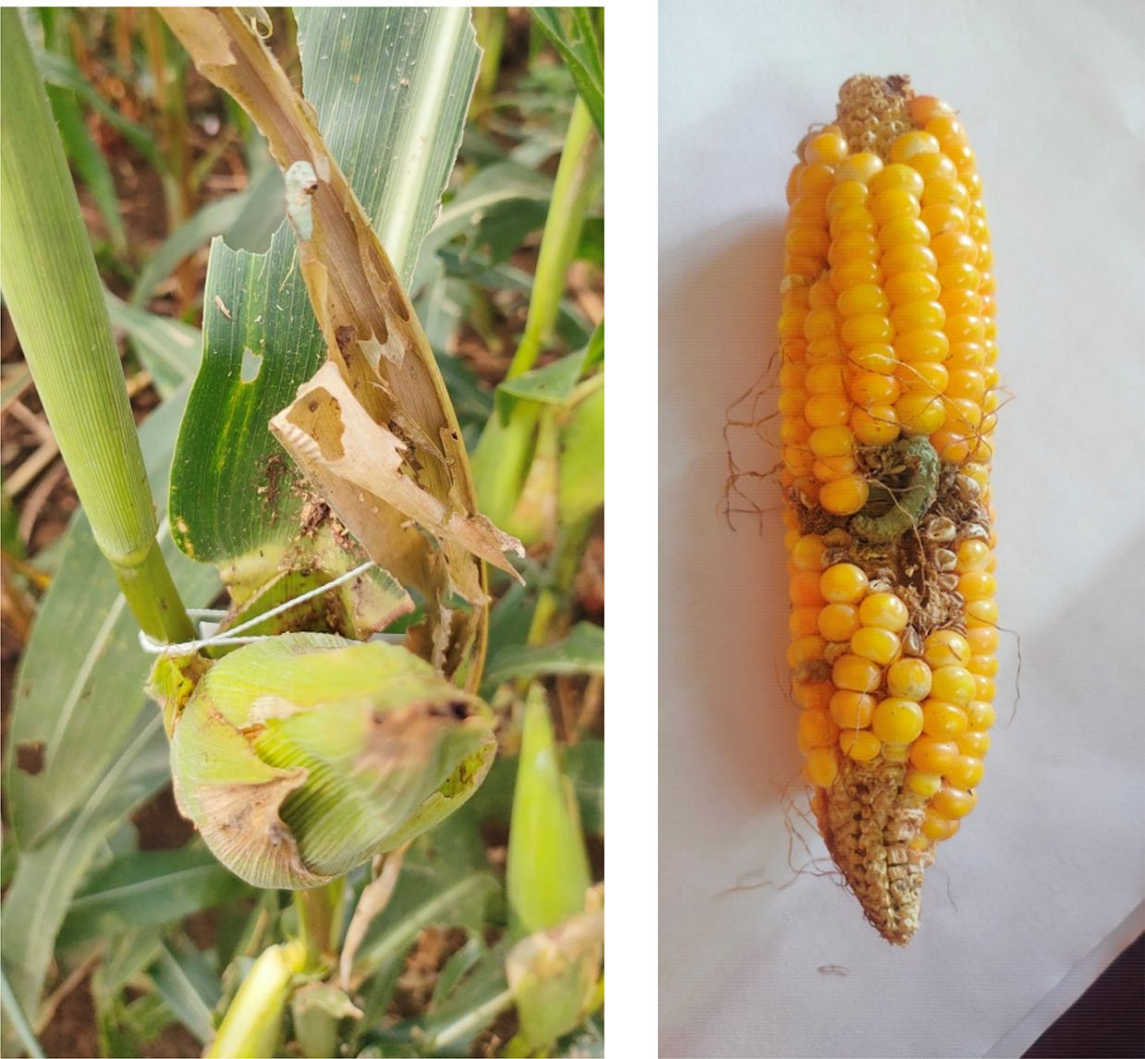
Mummification of *Metarhizium anisopliae* infected larvae of *S. frugiperda.*

The mean per cent infestation of *S. frugiperda*, before and after the application of different chemicals in IPM adopted villages during 2019–20, 2020–21 and 2021–22 were analyzed and presented in Tables 1, 2 and 3, respectively. During 2019–20, application of all three chemicals viz., Azadirachtin, Emamectin benzoate, and *Metarhizium anisopliae* in IPM-adopted villages led to a significant reduction in FAW infestation. Use of azadirachtin decreased mean infestation from 34.87 to 18.06% (*t value* = 7.06**). Similarly, application of *M. anisopliae* (Fig. 2) effectively brought the infestation from 35.50 to 18.68% (*t value* = 16.19**). The application of EB resulted in the most effective reduction of FAW infestation from 28.52 to 10.56 (*t value* = 11.64**). A similar trend in population regulation was observed during 2020–21. However, the per cent infestation was significantly higher. The application of Azadirachtin, emamectin benzoate and *M. anisopliae* resulted in a decrease of *S. frugiperda* infestation from 41.06, 52.84 and 48.55 to 20.01 (*t value* = 1.51**), 10.70 (*t value* = 18.24**) and 21.32 (*t value* = 19.60**), respectively. During 2021–22, the efficacy of Azadirachtin, emamectin benzoate and *M. anisopliae* in reducing the FAW infestation respectively as follows 45.56 to 20.21% (*t value* = 16.56**), 55.97 to 11.08% (*t value* = 16.56**) and 51.83 to 26.87% (*t value* = 16.66**).

**Table 1:** The mean percent infestation of FAW, *S. frugiperda* before and after application of different chemicals in IPM adopted villages during 2019–20.

Village	Azadirachtin			Emamectin benzoate			*Metarhizium anisopliae*		
	Before	After	*T statistics*	Before	After	*T statistics*	Before	After	*T statistics*
IPM1	34.45	19.27	5.59**	28.67	10.26	14.37**	36.2	15.7	14.07**
IPM2	28.9	11.26	6.56**	22.84	7.68	20.28**	34.8	18.6	9.47**
IPM3	41.28	22.18	5.09**	32.88	12.98	16.38**	35.9	16.8	12.10**
IPM4	33.89	18.22	3.78**	28.61	11.12	16.81**	34.6	18.7	8.26**
IPM5	31.78	18.19	4.15**	27.89	9.18	21.65**	35.1	19.6	8.18**
IPM6	38.9	19.26	4.23**	30.21	12.16	16.00**	36.4	22.7	7.03**
Mean	34.87	18.06	7.06**	28.52	10.56	11.64**	35.50	18.68	16.19**

**Samples are significantly different at both 5% and 1% level of significance.

**Table 2:** The mean percent infestation of FAW, *S. frugiperda* before and after application of different chemicals in IPM adopted villages during 2020–21.

Village	Azadirachtin			Emamectin benzoate			*Metarhizium anisopliae*		
	Before	After	*T statistics*	Before	After	*T statistics*	Before	After	*T statistics*
IPM1	40.45	20.27	10.79**	54.17	10.66	40.08**	46.2	20.5	13.25**
IPM2	36.78	16.26	14.01**	44.9	9.68	36.82**	48.9	21.9	12.98**
IPM3	43.44	23.43	9.58**	58.19	12.98	35.92**	50.7	18.2	18.99**
IPM4	46.89	22.67	11.65**	58.67	11.12	43.55**	51.3	25.1	11.11**
IPM5	38.87	18.19	12.55**	47.89	9.51	39.61**	44.8	20.8	12.62**
IPM6	39.9	19.26	11.69**	53.21	10.26	41.30**	49.4	21.4	13.93**
Mean	41.06	20.01	1.51**	52.84	10.70	18.24**	48.55	21.32	19.60**

**Samples are significantly different at both 5% and 1% level of significance.

**Table 3:** The mean percent infestation of FAW, *S. frugiperda* before and after application of different chemicals in IPM adopted villages during 2021–22.

Village	Azadirachtin			Emamectin benzoate			*Metarhizium anisopliae*		
	Before	After	*T statistics*	Before	After	*T statistics*	Before	After	*T statistics*
IPM1	43.89	19.29	12.56**	52.45	9.78	42.75**	52.5	31.1	7.85**
IPM2	39.78	20.16	10.44**	60.59	13.44	34.93**	51.8	26.9	10.39**
IPM3	46.44	21.43	12.08**	53.29	12.98	28.97**	50.7	24.6	11.25**
IPM4	44.49	20.17	13.01**	54.17	8.78	37.26**	52.2	27.3	10.06**
IPM5	48.87	19.89	15.92**	57.45	11.23	32.11**	50.9	22.7	13.15**
IPM6	49.9	20.31	15.36**	57.89	10.26	41.91**	52.9	28.6	9.22**
Mean	45.56	20.21	16.56**	55.97	11.08	16.56**	51.83	26.87	16.66**

**Samples are significantly different at both 5% and 1% level of significance.

**Figure 3:**
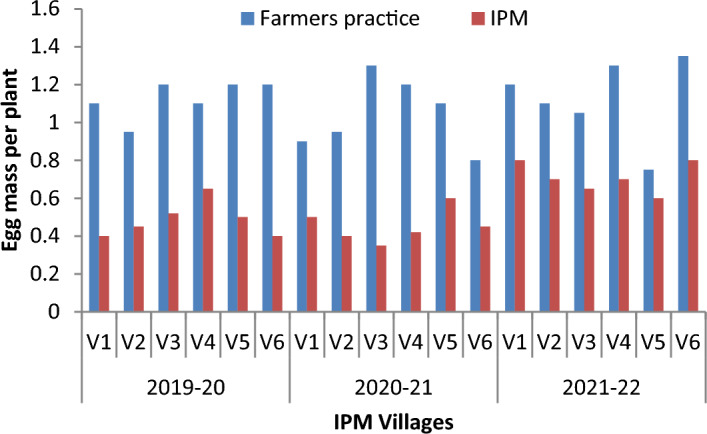
Mean number of egg mass/ plant observed in different villages (Mean of 5, 6, 7, 8 and 9 weeks observation).

**Figure 4:**
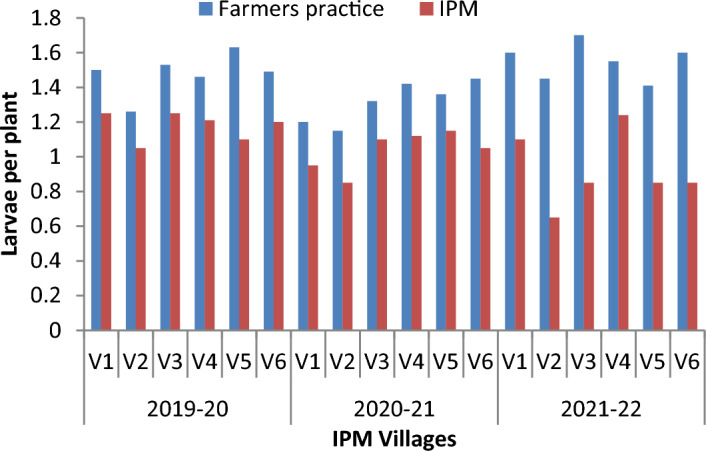
Mean number of larvae/ plant observed in different villages (Mean of 5, 6, 7, 8 and 9 weeks observation).

**Figure 5:**
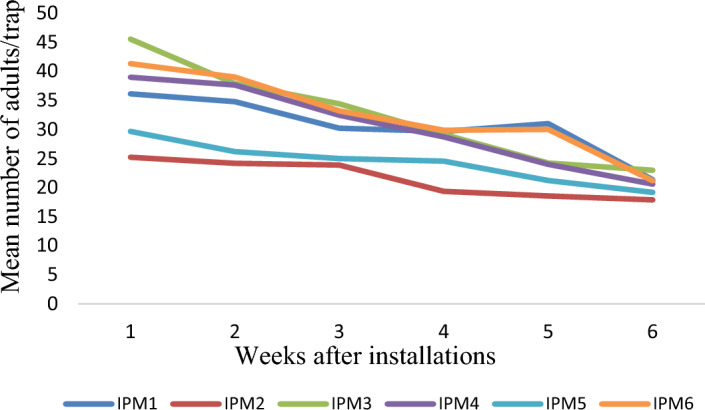
Mean number of adults/trap in different IPM villages during 2019–20.

**Figure 6:**
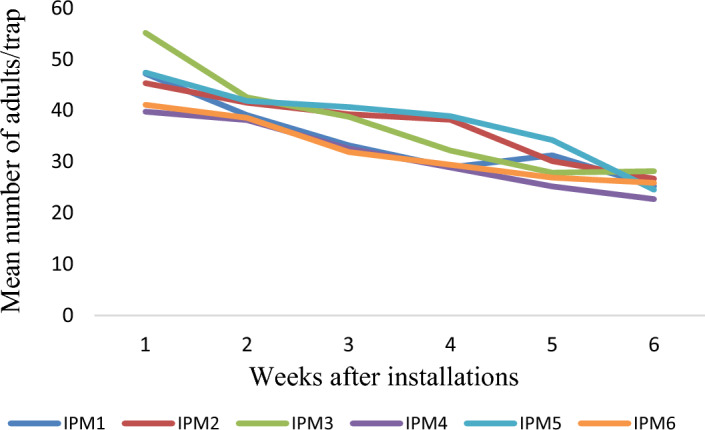
Mean number of adults/trap in different IPM villages during 2020–21.

**Figure 7:**
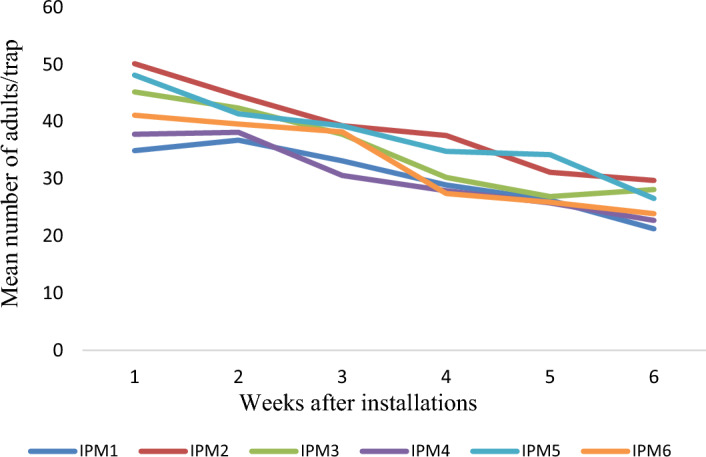
Mean number of adults/trap in different villages during 2021–22.

The results on number of egg mass and larvae per plant in different villages and years, comparing farmers' practice (FP) with IPM strategies, show that the egg mass/plant was lower in the IPM villages compared to the FP villages, indicating the effectiveness of IPM in reducing *S. frugiperda* infestation. The mean number of egg mass/plant on FP ranged from 0.95–1.2, 0.8–1.3 and 0.75- 1.35 during 2019–20, 2020–21 and 2021–22, respectively. However, the corresponding values in IPM villages show that the mean egg masses are 0.4–0.65, 0.4–0.6 and 0.6–0.8/ plant (Fig. 3). Similarly, the larvae/plant was significantly lower in IPM-adopted villages than in FP. The mean numbers of larvae per plant in FP villages are 1.26–1.63, 1.2–1.45 and 1.41–1.7, respectively, during 2019–20, 2020–21 and 2021–22. However, corresponding values on the mean number of larvae per plant in IPM-adopted villages are 1.05–1.25, 0.85–1.15 and 0.65–1.24 (Fig. 4). The results indicate the average trap catches in different IPM villages over the specified years. The results show that the trap catches varied among the different IPM villages across three years. There is some variation in trap catches within each village yearly. Interestingly, the adults trapped each week show a decreasing trend in all the observed locations across the years. The number of adults trapped each year ranged from 24.30–32.43, 31.21–37.94 and 30.20–38.72 months, respectively, during 2019–20 (Fig. 5), 2020–21 (Fig. 6) and 2021–22 (Fig. 7).

**Figure 8:**
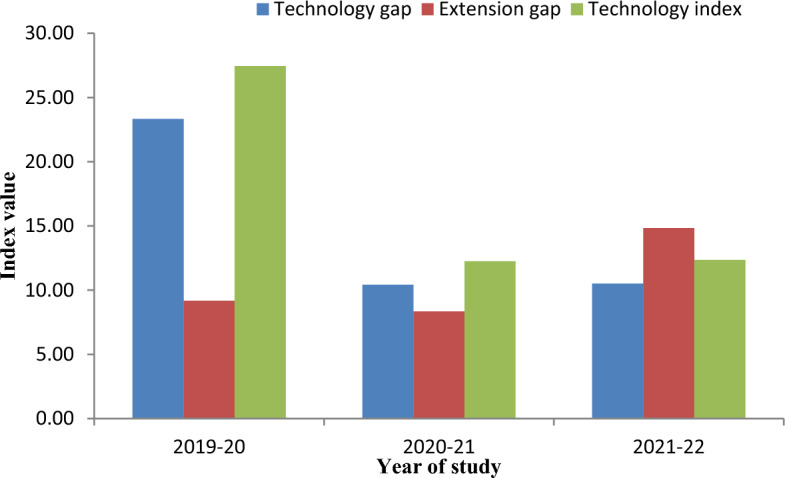
Technology gap, extension gap and technology index in adopting IPM strategies under demonstrated farmer’s fields. (Potential yield of maize = 85 q/ha in experimental area).

The technology gap, extension gap, and technology index in adopting Integrated Pest Management (IPM) strategies under the demonstration for three consecutive years (2019–20, 2020–21, and 2021–22) were presented in Fig. 8. In 2019–20, the technology gap was 23.33, representing the difference between the potential yield of maize (8500 kg (85 quintal)/ ha) and the actual yield obtained by farmers implementing IPM strategies. In 2020–21, the technology gap was reduced to 10.42. However, in 2021–22, the technology gap remained relatively stable at 10.50. Similarly, the extension gap was 9.17 during 2019–20, indicating the difference between the actual yield obtained by farmers implementing IPM strategies and the yield achieved under the demonstration. In 2020–21, the extension gap decreased to 8.33. However, in 2021–22, the extension gap increased to 14.82. The technology index was 27.45 in 2019–20, representing the extent farmers adopted and successfully implemented IPM strategies compared to the potential yield. In 2020–21, the technology index improved to 12.25. However, in 2021–22, the technology index slightly increased to 12.35.

The data shows that implementing IPM practices improved yields, increased gross and net returns, and favorable benefit–cost ratios compared to the traditional Farmer's Practice (FP). These results highlight the economic advantages of employing effective FAW management strategies in maize cultivation. In 2019–20, the adoption of IPM for FAW management (D) resulted in a higher yield of 61.67 q/ha compared to the FP yield of 52.5 q/ha. This represents a yield increase of 9.17 q/ha, translating to a percentage increase of 17.49% over the FP. The net return in the D scenario was USD 919.00, which was higher than the FP's net return of USD 659.96 (Table 4). Moreover, the B:C ratio for the D scenario was 2.74, indicating a favorable return on investment compared to the FP's ratio of 2.16. Moving to 2020–21, the D scenario continued to show positive results. The yield increased to 74.58 q/ha, with a yield increase of 8.33 q/ha (12.62% increase over the FP). The B:C ratio for the D scenario stood at 2.39, indicating a profitable outcome compared to the FP's ratio of 1.94. The D scenario demonstrated even greater success in the most recent year, 2021–22. The yield increased significantly to 74.49 q/ha, resulting in a substantial yield increase of 14.82 q/ha (24.87% increase over the FP), with the B:C ratio for the D scenario was 2.33 compared to the FP's ratio of 1.77 (Table 4).

**Table 4:** Economics of management of fall armyworm in maize during different growing years.

Year	Yield (q/ha)		Yield increase over FP	% increase over FP	Gross returns (USD)		Cost of cultivation (USD)		Net return (USD)		B:C ratio	
	FP	D			FP	D	FP	D	FP	D	FP	D
2019–20	52.5	61.67	9.17	17.49	1232	1447.11	572.04	528.11	659.96	919	2.16	2.74
2020–21	66.25	74.58	8.33	12.62	1387.67	1562.23	715	656.01	672.67	906.20	1.94	2.39
2021–22	59.675	74.49	14.82	24.87	1395	1741.52	790.99	748.59	604.00	992.93	1.77	2.33

Exchange rate used for conversion of INR to USD in the respective years.

## Discussion

The fall armyworm, *S. frugiperda*, is a devastating pest that threatened maize production within a year of its invasion in India and spread throughout the maize growing regions of India^15^. Owing to favorable climatic conditions and also a wide range of crops being grown in the Indian subcontinent, the impact of the FAW invasion in India is everlasting. The farmers have been grappling with the challenges posed by FAW infestation, which can lead to significant yield losses if not effectively managed. To manage this pest, Indian farmers have used various methods, including chemical insecticides. The data on farmer’s interviews highlights that most farmers (55%) prefer using pesticides as their primary approach for fall armyworm control. However, there is also some interest in alternative methods, such as pheromone traps and combining pesticides with cultural practices or biological agents. These findings underscore the need to promote sustainable pest management practices and provide farmers with information and resources to make informed decisions regarding FAW control methods.

Integrating IPM components resulted in the sustainable management of *S. frugiperda*. Installation of pheromone traps helps monitor and mass-trapping adults of fall armyworm^20^. The results of the present study shows that the pheromone trap catches varied between the different IPM fields, interestingly, the number of adults trapped decreased with each meteorological week. Further, the number of egg masses was also less in the IPM-adopted villages showing the efficacy of traps in bringing down both the adults and larval populations. Further, water-pan traps baited with different lures placed at 1.5 m and 2 m above the ground captured the highest number of moths^21^. They recommended it for decision-making regarding pest control. Cruz et al.^21^ found that the number of trap-caught moths was the best means of deciding on insecticide application in maize to control the fall armyworm. The reduction in egg masses seen in IPM fields is largely attributed to the amalgamation of compatible strategies. Notably, pheromone traps efficiently ensnare adult male moths, while the application of selective insecticides based on ETL effectively curtails future populations^21^. Moreover, the use of these selective insecticides poses minimal risk to natural enemies. Consequently, populations of natural enemies, particularly egg parasitoids, thrive, further diminishing egg masses. Remarkably, farmer training in the collection and destruction of egg masses significantly contributes to the decline of *S. frugiperda* in IPM-managed fields^22^. Moreover, the presence of bird perches and clean cultivation practices also played a role in reducing pest incidence within the IPM fields. FAW adults prefer to lay eggs in farmer practice fields despite the application of a cocktail of insecticides. It might be due to the farmers not adopting pheromone traps and differential egg placement *i.e.,* if the FAW populations are high or if the crop foliage remains soft (as during the first nine weeks of crop establishment), moths will lay eggs higher up on the plant or on surrounding vegetation^20^. In such cases, even if insecticides are applied directly to the crop, they may not effectively reach these higher areas where egg deposition occurs.

Applying azadirachtin 1500 ppm decreased mean infestation from 34.87 to 18.06, 41.06–20.01 and 45.56 to 20.21% during the study period. Azadirachtin is a naturally occurring compound with insecticidal properties derived from the neem tree (*Azadirachta indica*)^23^. It is commonly used in organic farming as a biopesticide and found to be effective against a variety of insect pests, including the FAW that infests maize crops. Tiwari and Boppu^24^ discuss various bio-safe alternatives for controlling FAW and recommended application methods of azadirachtin against FAW in maize. King and Ataholo^25^ also evaluated the effectiveness of azadirachtin 1500 ppm. They reported high efficacy against FAW larvae and recommended to be used as components for integrated pest management (IPM) plans for FAW under smallholder farmer conditions.

In our study, the efficacy of *M. anisopliae* on *S. frugiperda* was found effective in reducing the damage percentage throughout the investigation period, *i.e.,* from 35.50 to 18.68, 48.55 to 21.32 and 51.83 to 26.87%, respectively during the three consecutive years. Further, we observed a high level of incidence during November this is mainly due to the favorable weather conditions, which enables the rapid multiplication of *M. anisopliae.* Similar findings were reported by Kumar and Singh^4^ and Keerthi et al*.*^26^. Further, Sharma and Thakur^27^ explore the effectiveness of *M. anisopliae* against FAW and evaluate farmers' perception of this biopesticide. The study highlights the positive impact of using *M. anisopliae* on FAW control and the willingness of farmers to adopt this approach, emphasizing its potential as an eco-friendly and sustainable tool for FAW control. Fernandes et al*.*^28^ conducted field trials in Brazil, they found that a formulation of *M. anisopliae* applied alone or in combination with an insecticide effectively controlled FAW in maize. Elzen et al*.*^29^ investigated using *M. anisopliae* with a sex pheromone lure for fall armyworm control. They found that combining the fungal pathogen and the lure significantly reduced fall armyworm damage to maize plants. However, it is important to note that the actual effectiveness of this biological control agent can vary depending on various factors^30^, including climatic conditions^31^, application methods^32^ and population dynamics of the pest^33^.

In the present study, Emamectin benzoate was chosen purposefully as per the DPPQS made an ad-hoc recommendation and also, the farmers opined that it is giving a consistent result in reducing the incidence of FAW. Our results show that the incidence of FAW reduced from 28.52 to 10.56, 52.84 to 10.70 and 55.97 to 11.08% during the study period. The findings are in accordance with the study conducted by Wankhede et al*.*^34^ assessed the efficacy of different insecticides, including emamectin benzoate, and they found that Emamectin benzoate provided excellent control of the pest, with significantly higher grain yield compared to untreated control plots^35,36^. It is important to note that while emamectin benzoate is effective against fall armyworms, farmers need to follow recommended application rates and safety guidelines to minimize any potential negative impacts on the environment and non-target organisms^37^. The outcome of the farmer’s interview showed that the primary intention of applying insecticide is to achieve a higher yield. While synthetic insecticides play a role in pest management, relying solely on chemical control is unsustainable and can harm beneficial insects and ecosystems^38^. Initially, widespread use of synthetic pesticides was common in smallholder farming systems to combat FAW. However, farmers faced challenges with the effectiveness of these pesticides against FAW, leading to increased costs and risks associated with their intensive use^39^. To address these challenges, farmers need access to diversified integrated pest management strategies, improved extension services, and research-based recommendations tailored to their specific contexts. However, the results of the present study showed that adopting IPM resulted in maximum grain yield (61.67 q/ha) and monetary return (2.74 rupees/ rupee invested) compared to the sole application of insecticides. Reddy et al*.*^40^ reported that the benefit–cost ratio was significantly higher in the recommended approach (2.51) when compared to the farmer’s practice (2.12). The higher grain output and better market pricing of the produce may be the causes of the maize demonstration's higher net returns and B:C ratio^37,40^. The lower yield in the farmer’s field might be attributed to the use of insecticides, in which FAW known to developed resistance against most of them for instance cypermethrin^16^, lambda cyhalothrin^17^ and profenophos^18,19^. This is supported by the highest average number of egg masses and larvae per plant observed in the farmers practices examined in our study. The implementation of IPM techniques, such as mechanical removal and destruction of egg masses, trapping adult moths with pheromone traps, and employing self-sustaining entomopathogen alongside multi-modal azadirachtin, likely played a role in achieving the highest crop yields and net profits for the farmers^20,24,33^.

The technology index, as defined by Jeengar et al.^41^, serves as a measure of the suitability of advanced technology implementation in farmers' fields, with a lower score indicating higher feasibility. The collaboration of farmers in conducting such demonstrations, resulting in positive outcomes in subsequent years, is reflected in the fluctuating trend of the technology gap, ranging from 10.42 to 23.33. Variations in soil fertility, nutrient enrichment, organic manure application, and environmental factors such as rainfall and temperature, as noted by Dhandhalya and Shiyani^42^, likely contribute to observed discrepancies in the technology gap. Over the study period, there is a consistent upward trend in the extension gap, ranging from 9.17 to 14.32 q ha^−1^, highlighting the efforts of scientists to educate farmers on Integrated Pest Management (IPM) practices. Notably, a lower technological index value signifies greater practicality of technological implementation on farms. Throughout the experimental duration, the substantial fluctuation in the technological index, spanning from 12.25 to 27.4 percent, may be attributed to variances in soil fertility status and meteorological conditions.

## Methods

### Farmer’s interview and the study location

**Figure 9:**
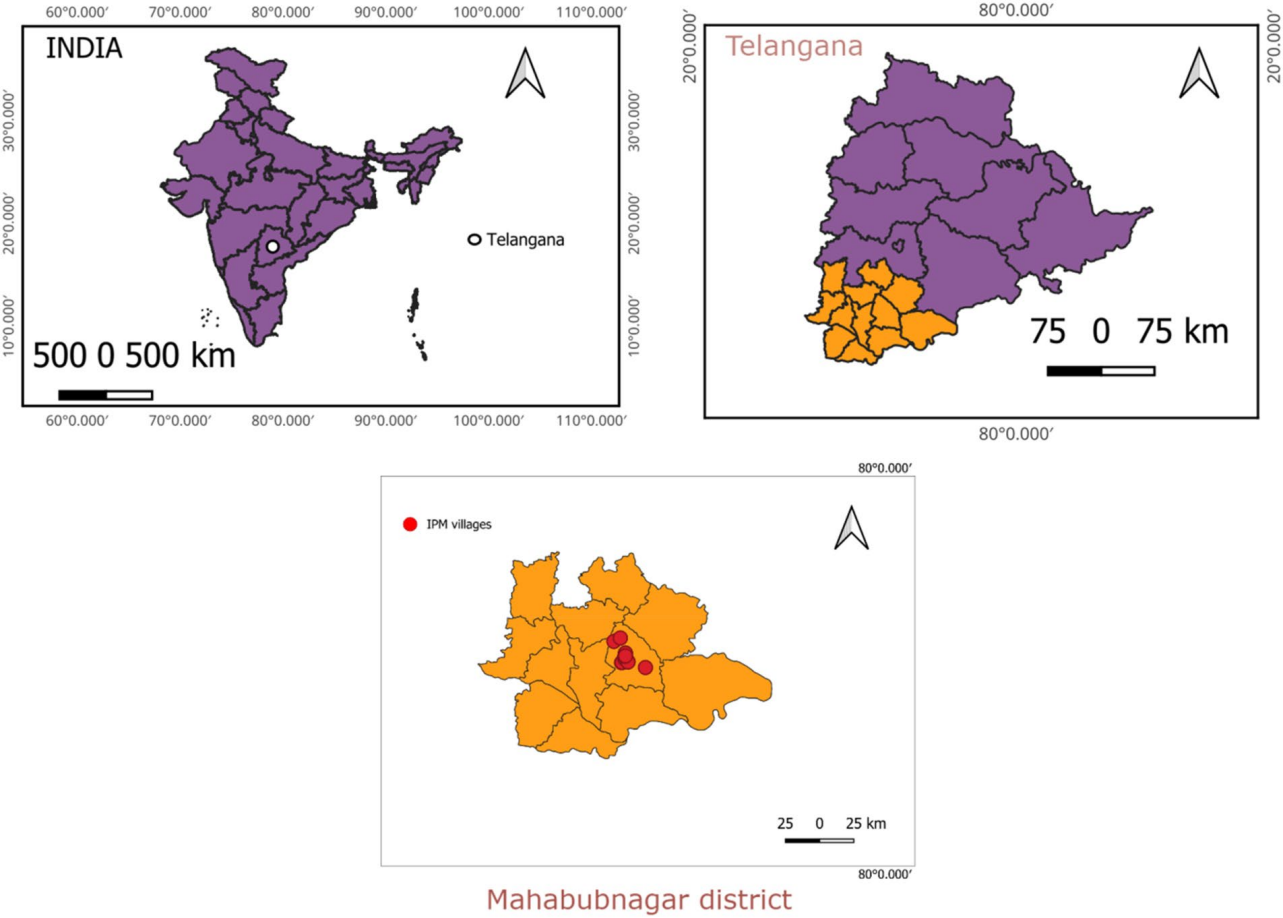
GIS maps depicting the study location and IPM practicing villages (Year 2019–20: IPM1—16.5078734, 78.231732; IPM2—16.5082880, 78.228844; IPM3—16.623484, 78.1588717; IPM4—16.6463967, 78.202408; IPM5—16.4793954, 78.210666; IPM6-n16.4460255, 78.372663. Year 2020–21: IPM1—16.480701596, 78.2149810; IPM2—16.48102895, 78.21498102, IPM3—16.48056361, 78.21398727, IPM4—16.54407863, 78.23688299, IPM5—16.54439100, 78.23664425, IPM6—16.54378814, 78.23687441. Year 2021–22: IPM1—16.5067690, 78.2334704; IPM2—16.5052449, 78.2334493; IPM3—16.4866143, 78.2544631; IPM4—16.4836707, 78.2544438; IPM5—16.5233138, 78.2327685; IPM6—16.5244808, 78.2370171.)

The farmers (n = 200) of study locations were interviewed (1-on-1) for their preference for pest management practices. The questionnaire used in the survey was provided in the supplementary material. We secured the necessary permissions for conducting the interview from our host institution, Professor Jayashankar Telangana State Agricultural University (PJTSAU), Telangana, India. The interviews were carried out in accordance with relevant guidelines and in alignment with the technical program approved by the institute research committee (IRC). Further, we also obtained informed consent from the farmers or their guardians before proceeding with the interviews. The selection of IPM components was determined by both the control methods preferred by farmers and the availability of biological inputs in the study area. The Krishi Vigyan Kendra (KVK), Palem, Nagarkurnool of PJTSAU, Telangana, India has conducted 18 On-farm Testing (OFTs) on “Assessment of IPM modules against invasive Fall armyworm, *S. frugiperda* in Maize”, under real irrigated farming situations during Rabi season for consecutive three years 2019–20, 2020–21 and 2021–22 at different adopted villages (n = 18) (Fig. 9). The GPS coordinate of the study locations provided with the figure. All 18 on-farm demonstrations were conducted in semi-arid areas of the southern Telangana zone of Telangana state, India. The main objective is to distribute and integrate the appropriate IPM knowledge to manage fall armyworm on maize effectively among all farmers.

### Treatment details

The farmer's meeting and diagnostic field visits to train the farmers were conducted during the crop period to create awareness of IPM modules to manage FAW effectively. Four IPM components were selected based on the farmers interview consisting of pheromone traps (8 traps with 40 lures *Spodoptera frugiperda* ® Pheromone chemicals, Hyderabad, Telangana, India), Azadirachtin 1500 ppm (Neem Gold ® Foliage Crop Solutions Private Limited, Chennai, Tamil Nadu, India) @ 1 L, Emamectin benzoate 5% SG (Proclaim ® Crystal Crop Protection Limited, Telangana, India) @ 100 g and *Metarhizium anisopliae* (® Biological control laboratory, PJTSAU, Rajendranagar, Hyderabad, Telangana, India) @ 1 kg/ acre. The chosen farmers received guidance and practical demonstrations on various topics, such as installing pheromone traps and applying plant protection chemicals at the appropriate times based on the ETL level of the pest (Table 5). The procedures, including the selection of sites and farmers, the establishment of demonstration layouts, and active farmer participation, were implemented in accordance with the methods given by Choudhary^43^. Each IPM module and farmer's practice covered an area of 0.4 hectares (equivalent to 1 acre), and a minimum of 3 villages with a total of 6 locations. The treatment plan comprises several key components: the deployment of pheromone traps starting from the early vegetative stage (when the crop is 7 days old) and continuing until harvest, with lure replacement every 21 days. Additionally, Azadiractin at 1500 ppm and Emamectin benzoate at 5% SG are applied when the pest population reaches the Economic Threshold Level (ETL). ETL is stage specific and the ETL thresholds for *S. frugiperda* are as follows: (a) 1–2 larvae per whorl, (b) 5% of seedlings being cut, (c) 15% of whorls infested in young plants within the first 30 days, and (d) Initiation of management practices if pheromone traps capture more than 2–3 adult moths for three consecutive days^44^.

**Table 5:** Information on treatments.

On Farm Testing/ IPM practices	Farmers practice
1. Avoid staggered sowing of maize	1. Staggered sowing of maize
2. Installation of pheromone traps @ 8 per acre	2. Not installing pheromone traps
3. Clean cultivation balanced application of fertilizers	3. No application of Azadiractin 1500 PPM at the early stage of the crop
4. Erection of bird perches @10/acre	4. Spray of following synthetic pesticides twice at weekly interval
5. Mechanical/ manual collection and destruction of egg masses	5. Initial spray with Profenophos 50% EC @400 ml/acre, cost is: 680/-
6. Spraying of Azadiractin (1500 ppm) to deter and repel the egg laying of FAW	6. 2nd spray is with Lambda cyhalothrin 5% EC@ 250 ml/acre, cost is: 590/-
7. Trap catches > 3 adult moths for three consecutive days need based whorl application of Emamectin benzoate 5% SG @ 0.5 g/L of water	7. 3rd spray with Chlorantraniliprole 18.5% SC @ 60 ml/acre, cost is: 920/-
8. Spraying with bio fungicide *Metarhizium anisopliae* @ 5 g/L of water	8. 4th spray with Cypermethrin 25% EC @ 100 ml/ acre, cost is: 490/-

In order to ensure the effectiveness of bio-fungicide, *Metarhizium anisopliae* (5 g/L), farmers who have adopted this approach were advised to abstain from any other synthetic chemical spraying for at least two weeks when facing severe FAW infestations. This is because *M. anisopliae* has a self-propagating nature and exhibits effective dispersal abilities, making it a reliable choice in such circumstances. Furthermore, farmers underwent training sessions aimed at identifying egg masses. This involved meticulous inspection of maize plants, wherein they searched for egg masses on the leaves or stems. This inspection was conducted by walking in a "W" pattern across the field, leaving out 3–4 outer rows. As they proceeded along the first straight line, they selected groups of 10 plants and tallied the number of egg masses. This counting method continued in a “W” fashion, gathering data from at least 40 plants. Additionally, they were advised to examine damaged whorls and record the number of larvae present in infested whorls at 15-day intervals^45^. Both expressed in mean egg mass/ plants and mean larvae/ plants, respectively. In contrast, the farmers’ practice includes spraying synthetic pesticides (Table 5) twice weekly. For duration of three years, all essential inputs were consistently distributed to each farmer as part of a demonstration^46^. Observations on the incidence of FAW were made based on the number of whorl damage in 50 randomly selected maize plants^47,48^. The whorls were manually opened and inspected for FAW larvae. The infestation ratio was determined by counting infected whorls and then represented as a percentage. The total number of male adult moths in each trap was counted at weekly intervals and later presented as a mean number of moths trapped up to crop harvesting. To evaluate the bioefficacy of azadirachtin, emamectin benzoate and *M. anisopliae*, the number of FAW larvae in each 50 m^2^ area (minimum of 50 plants) was counted before and after the application of insecticides. FAW incidence before and after treatment of pesticides and some plant protection sprays was recorded in On Farm Testing (OFT)/ IPM practicing and non-practicing farmer's field plots^49^. The per cent reduction in pest population was calculated using

$${\text{Percent}}\;{\text{population}}\;{\text{reduction}} = \left( {{\text{X}}_{{\text{i}}} - {\text{X}}_{{\text{o}}} } \right)/{\text{X}}_{{\text{i}}} \times 100$$

where X_i_—number of larvae before insecticide application and X_o_—number of larvae after insecticide application^50^. The two-sample t-test was performed to study the significant difference between the before and after application of respective biopesticides, and insecticides using an online statistical software WASP-Web Agri Stat Package 2.0 (https://ccari.icar.gov.in/wasp2.0/index.php).

### Economics of IPM module

The economics of IPM module and farmers practice were worked and qualitative data were converted into quantitative form and expressed in terms of per cent increase in yield^40^. Finally, the extension gap, technology gap, technology index along with benefit cost ratio was worked out^51,52^ by using following formula:

$${\text{Technology gap }} = {\text{ Potential yield }}{-}{\text{ Demonstration Yield}}$$

$${\text{Extension}}\;{\text{gap}} = {\text{Demonstration}}\;{\text{yield}} - {\text{Farmers}}\;{\text{yield}}$$

$${\text{Technology}}\;{\text{index}} = \frac{{{\text{Potential}}\;{\text{yield}} - {\text{Demonstration}}\;{\text{yield}}}}{{{\text{Potential}}\;{\text{yield}}}} \times 100$$

### Ethics approval

The collection of insects and insect data, as well as experimental research and field studies on integrated pest management (IPM) techniques were conducted in accordance with pertinent institutional, national, and international regulations. The required necessary permissions were obtained from the host institution, Professor Jayashankar Telangana State Agricultural University (PJTSAU), Telangana, India for conducting the interview.

## Conclusion

Field demonstrations conducted over three consecutive years in semiarid regions of India revealed the efficacy of a four-component IPM approach in significantly reducing FAW infestations compared to conventional practices reliant solely on insecticide applications. Notably, IPM implementation led to substantial reductions in egg mass and larvae infestations, highlighting its effectiveness in curbing FAW populations at different growth stages. IPM consistently outperformed traditional Farmer's Practices (FP) in reducing egg masses and larvae per plant. Moreover, adopting IPM led to increased yields (9.17, 8.33 and 14.82% over farmers practice), gross and net returns, and favourable benefit–cost ratios (2.74, 2.39, and 2.33), reinforcing the economic benefits of strategic FAW management in maize cultivation. These findings underscore the significance of IPM in enhancing agricultural sustainability and profitability. For effective management of FAW in maize crops, government policies primarily revolve around implementing preventive measures, providing farmers with the necessary information, and supporting research and development efforts. Cooperative efforts between agricultural extension services and farmers can help build trust, provide ongoing support, and address any concerns or challenges farmers may face while implementing the IPM strategies against *S. frugiperda* in India.

### Supplementary Information

Supplementary Information.

## Data Availability

All data have been provided in the manuscript.
