# Supplementary material for: Evaluation of farmers friendly IPM modules for the management of fall armyworm, Spodoptera frugiperda (JE Smith) in maize in the hot semiarid region of India
Source: Sci Rep. 2024 Mar 26;14:7118. doi: 10.1038/s41598-024-57860-y (PMC10966016; doi:10.1038/s41598-024-57860-y)
Supplement: Supplementary file 1 — Supplementary Information. [file 41598_2024_57860_MOESM1_ESM.pdf]

Entomological Society of India, Division of Entomology, ICAR- IARI, New Delhi. Survey Proforma for Farmers' Perception on Fall Armyworm, *Spodoptera frugiperda* (J.E. Smith) Invasion in India

Farmer No.			

A: Farmer identification

Q. No.	Farmer identification	Response	Q. No.	Farmer identification	Response
A01	Village		A09	No of years of formal education	
A02	Block		A10	No of years of farming experience	
A03	District		A11	Family size (Numbers)	
A04	Mobile no.		A12	Membership in farmer organization/cooperative (Yes=1, No=2)	
A05	Name of the farmer		A13	Do you receive income from non-farm sources? (Yes=1, No=2)	
A06	Gender (Male=1, Female=2)		A14	Total farm land owned (ha)	
A07	Age (Years)		A15	Total livestock units owned (number of farm animals owned)	
A08	Category (General=1, SC/ST=2, OBC=3, Others=4)		A16	Do you have attached labour for farm operations? (Yes=1, No=2)	
	Distance to the nearest agro-dealer (km)			Distance to the nearest agricultural extension office (km)	

B: Cropping pattern

Crop	Variety	Variety type (1=Local, 2= HYV, 3=Hybrid)	Area planted (hectares)	Quantity of seed used (kg)	Seed price (Rs/ kg)	Season (1=Kharif, 2=Rabi, 3=Summer)
**Maize**						
**Sorghum**						

C: Crop production and inputs (kg)

Crop	Crop production (kg)	Chemical fertilizer quantity (kg)	Chemical fertilizer expenditure (Rs)	Organic/ Bio fertilizer (Rs)	Pesticide/ Insecticide (Rs)	Labour (Rs)
**Maize**						
**Sorghum**						

D: Farmers' awareness about Fall Army Worm (Yes/No)

Sl. No	Question	Response (Yes=1, No=2)
1	Are you aware about FAW?	
2	Have you observed infestation of FAW in your field?	
3	If yes then at which stage:	
	Egg	

	Larva	
	Pupa	
	Adult	
4	On which crop you have noticed the damage of FAW	
	Maize	
	Sorghum	
	Sugarcane	
	Pulses	
	Other cereals	
	Vegetables	
5	Whether you know the origin of pest?	
6	Can you differentiate the damage due to FAW and other *Spodoptera* sp.?	
7	Did you practice any control measures for FAW?	
8	I use my own sprayer for taking up control measure	

E. Farmers' response to FAW awareness statements (5 Point Likert Scale)

Sl No	FAW awareness statements	Strongly agree	Somewhat agree	Don' t know/Neutral	Somewhat disagree	Strongly disagree
1	FAW can be easily identified					
2	FAW invasion has made maize/sorghum cultivation difficult					
3	FAW invasion has reduced my crop yield					
4	Severity of *S. frugiperda* attack on maize/sorghum leaves is very high					
5	Severity of *S. frugiperda* attack on maize/sorghum ear is very high					
6	Spread of FAW increases with time in the cropping season					
7	FAW invasion cannot be controlled without taking proper control measures					
8	Controlling FAW invasion is very difficult					
9	I use seed treatment to manage FAW					
10	The cost of FAW control measures is very high					
	The existing FAW control measures are not effective					
11	I prefer to change crop to avoid FAW infestation					
12	I get sufficient information on FAW from public extension agencies					
13	I get sufficient information on FAW from private extension agencies/ SHGs					
14	I get sufficient information on FAW from ICT (TV/Radio/News paper/ Internet/ Mobile etc)					

15	I get sufficient information on FAW from other farmers					
16	I have attended training on FAW management					

F: Famer perception about severity of damage to his crop area

a) A very minor part (<10% area infested)
b) A minor part (10% to 40% area infested)
c) About a half (41% to 60% area infested)
d) A major part (61% to 90% area infested)
e) The entire area (>90% area infested)

G: Which method of control you mostly preferred for the management of FAW:

a) Pesticides
b) Biologicals
c) Use of pheromone trap
d) Physical/cultural practices
e) Pesticides + cultural practices
f) Pesticides + biologicals
g) Cultural + biologicals

H: Which of the following practices were used for FAW control

**Chemical measures**			**Physical/ cultural measures**	
Insecticide	***Frequency of application***	***Rate of application***	***Strategy followed***	***Frequency (where ever applicable)***
Emamectin Benzoate			Hand picking caterpillars/egg masses	
Chlorantraniliprole			Sand/ash	
Lambda Cyhalothrin			Early planting	
Thiamethoxam			Soil fertility management	
Indoxacarb			Destroying infected plants	
Spinetoram			Crop rotation	
Flubendiamide			Intercropping	
Novaluron				
Any other				

I: In your opinion which is the most effective practice to control FAW
